# Bilateral Superior Semicircular Canal Dehiscence Presenting as Progressive Gait Instability in a Patient With Parkinsonism and Autoimmune Features

**DOI:** 10.7759/cureus.103368

**Published:** 2026-02-10

**Authors:** Radmanesh Khalili, Shivangi Jha, Lukacs S Lesinszki, Haiyang Jiang, Peter G Bernad

**Affiliations:** 1 Neurology, Karl Landsteiner University of Health Sciences, Krems an der Donau, AUT; 2 Medicine, University of Szeged, Szeged, HUN; 3 Physiology, Semmelweis University, Budapest, HUN; 4 Neurology, Tongji Medical College, Huazhong University of Science and Technology, Wuhan, CHN; 5 Neurology, Neurology Services, Inc., Washington, D.C., USA

**Keywords:** atypical gait disturbance, corticosteroid responsiveness, diagnostic overshadowing, parkinson's disease, superior semicircular canal dehiscence

## Abstract

Superior semicircular canal dehiscence (SSCD) is a vestibular disorder caused by a defect in the bony roof of the superior semicircular canal, resulting in a third mobile window in the inner ear. This structural abnormality alters vestibular and auditory processing, producing symptoms such as vertigo, imbalance, sound sensitivity, autophony, and oscillopsia. We describe a 65-year-old man with a longstanding diagnosis of atypical Parkinsonism who presented with progressive gait apraxia, disequilibrium, and cognitive slowing. Despite undergoing bilateral deep brain stimulation (DBS) for tremor, his axial symptoms worsened. High-resolution computed tomography (CT) imaging later revealed bilateral SSCD. The patient reported subjective improvement during short courses of corticosteroids prescribed for unrelated autoimmune conditions. While objective physiological testing and lab markers were not available, the case underscores the risks of diagnostic overshadowing in neurodegenerative conditions and emphasizes the importance of identifying concurrent, potentially treatable vestibular pathology.

## Introduction

Superior semicircular canal dehiscence (SSCD), first described by Minor et al. in 1998, results from a bony defect in the roof of the superior semicircular canal, creating an abnormal third mobile window in the labyrinthine system and altering vestibular and auditory physiology [1]. Clinically, SSCD presents with a wide spectrum of auditory and vestibular symptoms, including sound- or pressure-induced vertigo (Tullio and Hennebert phenomena), autophony, oscillopsia, motion-provoked dizziness, and chronic imbalance [2]. These symptoms are often grouped under the term "third window syndrome", with SSCD being its most common cause.

Although many patients with SSCD exhibit symptoms localized to one side, up to 50% demonstrate bilateral anatomic dehiscence on imaging [3]. In unilateral cases, clinical signs such as nystagmus, sound- or pressure-induced vertigo, and vestibular evoked myogenic potentials (VEMPs) typically localize to the affected ear. In bilateral cases, however, these signs often remain lateralized, with nystagmus corresponding to excitation or inhibition of the superior canal on the side of stimulation. Notably, patients with bilateral dehiscence frequently show asymmetry in physiological measures, including larger air-bone gaps and lower cervical VEMP thresholds on the more symptomatic side, supporting a lateralized functional dominance despite bilateral structural pathology [4].

Recent studies have also described SSCD as a potential mimic of psychiatric or cognitive disorders, with patients reporting symptoms such as derealization, cognitive overload, panic-like episodes, and fatigue [5]. These presentations, particularly in individuals with Parkinsonism or atypical movement disorders, may lead to diagnostic overshadowing, where new symptoms are mistakenly attributed solely to central neurodegeneration.

Here, we present a patient with atypical Parkinsonism whose progressive axial and cognitive symptoms were ultimately explained by bilateral SSCD. This case highlights the importance of avoiding attribution bias and maintaining a broad differential diagnosis in complex neurological presentations.

## Case presentation

Early course and deep brain stimulation (DBS) intervention

A 65-year-old man with a 15-year history of tremor-predominant Parkinsonism began experiencing sit-to-stand initiation difficulties in 2019. Once upright, his stride and cadence were preserved, and his wife noted that he often walked faster than she could keep up, which was inconsistent with typical Parkinsonian freezing. These episodes responded to external cues such as light touch, suggesting functional gait apraxia rather than true akinesia.

Later that year, he underwent bilateral subthalamic nucleus deep brain stimulation (STN-DBS) for medication-refractory tremor. The procedure significantly improved his tremor and allowed the discontinuation of levodopa, but did not address his persistent axial instability. Gait and posture remained unpredictable, and freezing episodes worsened in sensory-rich environments. No Movement Disorder Society-Unified Parkinson's Disease Rating Scale (MDS-UPDRS) scores or detailed neuroimaging reports were retrievable from prior institutions, and this limitation is acknowledged.

Symptom progression and diagnostic uncertainty

By 2020, he developed increasingly atypical features, including cognitive slowing, emotional lability, and difficulty communicating in noisy environments. He reported increasing intolerance to visually complex or acoustically crowded settings, often describing sensations of "overload". Between 2020 and 2024, his caregivers documented progressive hypomimia, stooped posture, and sensory hypersensitivity.

Despite preserved gait once walking began, initiating movement became more unpredictable and effortful. His constellation of symptoms led to divergent diagnostic interpretations across external institutions. Some clinicians favored atypical Parkinsonism, others proposed an autoimmune or inflammatory process, and still others attributed the findings to advanced Parkinson's disease. The absence of consistent subtype labeling further reflected diagnostic uncertainty.

During these years, the patient received multiple short courses of corticosteroids for thyroid- and adrenal-related conditions. He and his caregivers consistently reported subjective improvement in dizziness, oscillopsia, and sensory overload during these intervals. Because these observations lacked physiologic correlation, they are reported strictly as patient-perceived improvements rather than as evidence of steroid-responsive vestibular disease.

Diagnostic breakthrough: identification of bilateral SSCD

**Figure 1:**
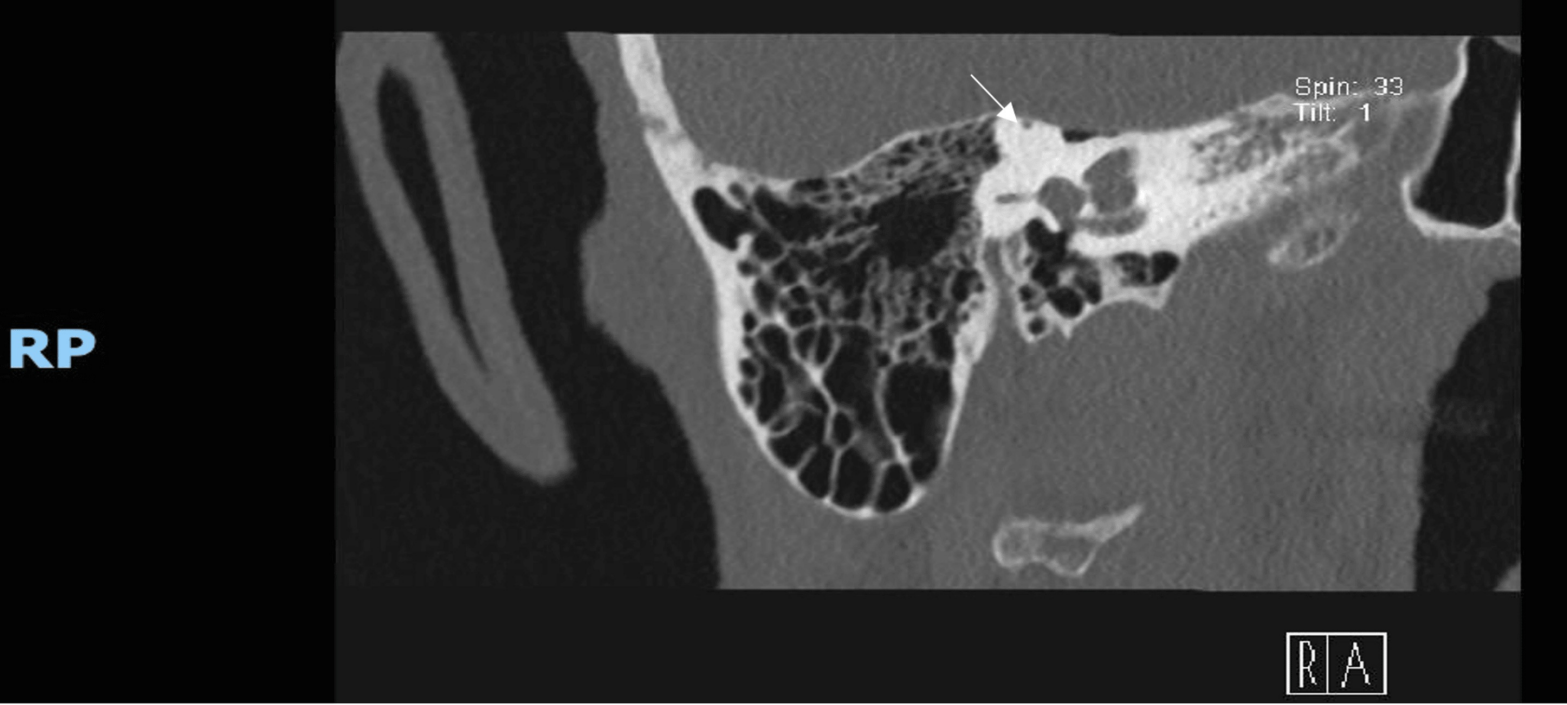
High-resolution coronal CT of the right temporal bone demonstrating clear absence of bony covering over the superior semicircular canal, consistent with right-sided superior canal dehiscence. Note the proximity of the dehiscent canal to the middle cranial fossa, confirming anatomical vulnerability typical of SSCD CT: computed tomography; SSCD: superior semicircular canal dehiscence

**Figure 2:**
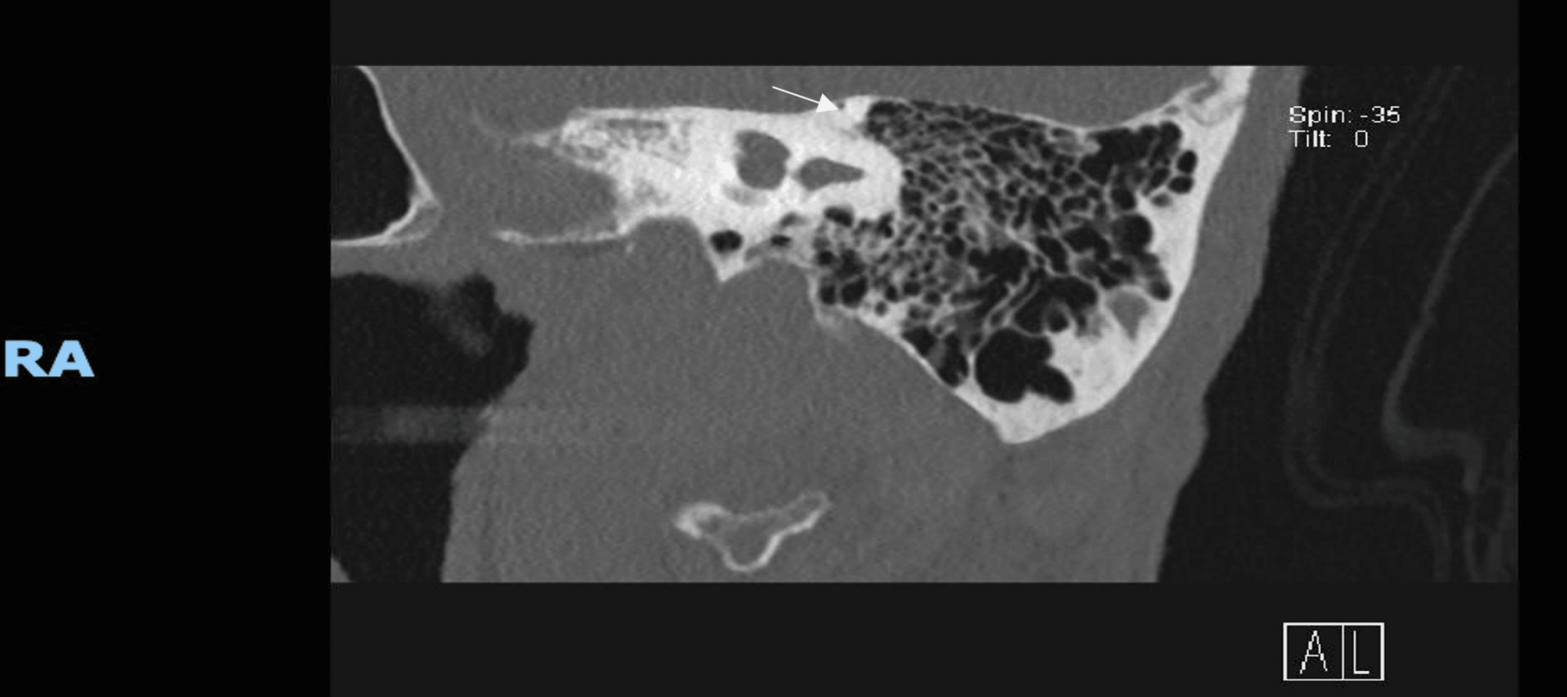
Coronal high-resolution CT of the left temporal bone revealing similar dehiscence of the superior semicircular canal, confirming bilateral involvement. Note the continuity of the membranous canal with the cranial cavity, suggestive of dual-sided "third window" physiology CT: computed tomography

In July 2025, high-resolution temporal bone computed tomography (CT) revealed bilateral SSCD, with clear dehiscence of the canal roofs on both sides (Figures 1-2). Reconstructions in targeted planes, including the Pöschl view, enhanced visualization of the defects and increased diagnostic confidence [5]. These imaging findings prompted renewed scrutiny of symptoms long attributed to Parkinsonism. Features such as autophony, oscillopsia, motion-provoked dizziness, and sound-triggered disequilibrium emerged as consistent with bilateral SSCD rather than exclusively central pathology.

Unfortunately, objective vestibular physiologic testing (e.g., cVEMP/oVEMP thresholds, vHIT, and audiometry) and reformatted CT images in the Pöschl or Stenvers planes were not available. These are noted as important diagnostic limitations. Nevertheless, the combination of clinical features and imaging led to a confident diagnosis of bilateral SSCD.

Comorbidities

The patient had a significant medical history of hypothyroidism following thyroidectomy, adrenal insufficiency, paroxysmal atrial fibrillation, and recurrent deep vein thrombosis. During treatment for his endocrine conditions, he received intermittent corticosteroid therapy (e.g., oral prednisone), during which caregivers noted temporary improvements in balance and mental clarity. Though anecdotal and undocumented in quantitative terms, this steroid responsiveness raised the possibility of an inflammatory or autoimmune component. However, lab results (including erythrocyte sedimentation rate (ESR), C-reactive protein (CRP), antiphospholipid antibodies, and encephalitis panels) from external evaluations were not accessible.

Outcome and reframing

As of late 2025, the patient had been referred to the Neurotology department at the University of California, Los Angeles (UCLA) for surgical consideration. At the time of final documentation, surgical repair had not yet been performed. Vestibular rehabilitation and environmental adjustments were initiated to improve safety and reduce sensory triggers.

Importantly, the diagnosis prompted a conceptual reframing of his clinical course. Rather than attributing all progressive symptoms to Parkinsonian decline, clinicians recognized the contribution of a structural vestibular disorder superimposed upon an underlying neurodegenerative condition.

## Discussion

This case illustrates the complex interplay between neurodegenerative and vestibular disorders. The patient's clinical picture, namely, axial dysfunction, postural instability, cognitive slowing, and hypersensitivity, initially conformed to atypical Parkinsonism. However, the failure of STN-DBS to address these features, and the presence of classic SSCD symptoms (autophony, oscillopsia, Tullio phenomenon), ultimately prompted re-evaluation. Recognition of bilateral SSCD reframed what had been perceived as neurodegeneration into a treatable vestibular condition.

Although anatomical bilateral SSCD is not uncommon, bilateral symptomatic SSCD is far rarer and more likely to cause severe disequilibrium and hypersensitivity [6]. The patient's subjective improvement during corticosteroid treatment, prescribed for unrelated endocrine conditions, also raises the question of autoimmune vestibulopathy. While a formal diagnosis of autoimmune inner ear disease (AIED) could not be made due to insufficient audiological or laboratory data, the overlap is conceptually important. Given the patient's history of thyroid disease, adrenal insufficiency, and thrombosis, systemic autoimmunity remains a plausible cofactor.

The absence of VEMP thresholds, audiometry, or reformatted imaging data presents diagnostic limitations. Nevertheless, the strong correlation between symptoms and imaging, along with reproducible bedside testing, justifies the diagnosis of bilateral SSCD.

Additionally, this case emphasizes a known limitation of STN-DBS: while highly effective for tremor and bradykinesia, DBS often fails to resolve gait or axial impairments [7]. Persistent postural symptoms after effective tremor control should prompt the reconsideration of other diagnoses, including third window disorders such as SSCD.

## Conclusions

Bilateral SSCD can mimic or exacerbate central neurologic symptoms and should be considered in patients with coexisting Parkinsonism who present with atypical features, such as preserved gait mechanics despite freezing, hypersensitivity to auditory or visual stimuli, or apparent cognitive slowing in complex environments. Although objective vestibular and serologic data were unavailable, the patient's clinical history and imaging findings support the diagnosis. Recognition of SSCD led to a paradigm shift in management and a potential surgical treatment path. Clinicians should be alert to overlapping sensory disorders, especially in cases where conventional neurodegenerative treatment fails or symptoms evolve atypically.
